# cGAS Regulates the Radioresistance of Human Head and Neck Squamous Cell Carcinoma Cells

**DOI:** 10.3390/cells11091434

**Published:** 2022-04-23

**Authors:** Taichi Nyui, Hironori Yoshino, Tetsuya Nunota, Yoshiaki Sato, Eichi Tsuruga

**Affiliations:** 1 Department of Radiation Science, Hirosaki University Graduate School of Health Sciences, Hirosaki 036-8564, Aomori, Japan; h21gg205@hirosaki-u.ac.jp (T.N.); h20gg702@hirosaki-u.ac.jp (Y.S.); tsuru@hirosaki-u.ac.jp (E.T.); 2 Department of Radiological Technology, Hirosaki University School of Health Sciences, Hirosaki 036-8564, Aomori, Japan; tetsuya.123654@gmail.com

**Keywords:** cyclic GMP-AMP synthase, head and neck squamous cell carcinoma, radiosensitivity, apoptosis, mitotic catastrophe, cellular senescence, p21

## Abstract

Cyclic GMP-AMP synthase (cGAS) plays an important role in biological responses to pathogens. The activation of the cGAS pathway in immune cells is known to induce antitumor effects, but the role of cGAS in cancer cells remains poorly understood. In silico analysis using public databases suggested that high cGAS expression in head and neck squamous cell carcinoma (HNSCC) is indicative of a poor prognosis for HNSCC patients. We therefore investigated the role of cGAS in malignancies and the cellular radiation response of human HNSCC cells (SAS and Ca9-22) in vitro, because radiotherapy is one of the treatments most commonly used for HNSCC. Although cGAS knockdown failed to suppress the proliferation of non-irradiated HNSCC cells, it enhanced the radiosensitivity of HNSCC cells. The administration of the cGAS agonist increased the radioresistance of HNSCC cells. cGAS knockdown increased radiation-induced mitotic catastrophe, apoptosis, or cellular senescence, depending on the cell line, and this cell line-dependent response might be due to different responses of p21 after irradiation. Collectively, our findings indicate that the cGAS pathway regulates the radioresistance of HNSCC cells.

## 1. Introduction

Cancer is a global problem, affecting the health of millions of people worldwide. According to the WHO Global Cancer Observatory (GLOBACAN), about 19 million new cases of cancer were diagnosed in 2020, and almost 10 million people died from the disease [1]. The Global Burden of Diseases, Injuries, and Risk Factors Study reported 100 million cases in 2017, a 1.59-fold increase from 1990 [2]. Although remarkable progress has been made in cancer treatment in recent years including the development of targeted molecular therapy and radiotherapy [3,4], the existence of treatment-resistant cancer cells such as radioresistant cancer cells produces a poor prognosis for cancer patients [5,6,7,8].

Cyclic GMP-AMP synthase (cGAS) is a pattern recognition receptor that recognizes pathogen-derived components and induces a biological response to pathogens. In cells infected with pathogens such as DNA viruses, cGAS recognizes pathogen-derived double-stranded DNA in the cytoplasm and synthesizes cyclic GMP-AMP (cGAMP) [9,10]. The synthesized cGAMP acts as a second messenger, and activates Stimulator of Interferon Genes (STING), a molecule localized in the endoplasmic reticulum. STING eventually activates the transcription factor interferon regulatory factor 3 (IRF3) and nuclear factor-kappa B [11,12]. As a result of the activation of these transcription factors, cytokines such as type I Interferon (IFN) are produced, and eliminate pathogens.

Recent studies have shown that the cGAS-STING pathway is closely related to cancer development and progression [13,14]. For example, 7,12-dimethylbenz(a)anthracene has been shown to promote skin tumorigenesis through STING activation in a mouse model [15]. It has been reported that the prognosis of patients with lung adenocarcinoma with high levels of cGAS is better than that of patients with low levels, while breast cancer patients with high levels of cGAS have poor prognoses [16]. These findings suggest that cGAS may be a prognostic marker for certain cancers. However, it is known that the cGAS-mediated immune response is important for the effectiveness of cancer therapy. Self-DNA released from tumor cells treated with cancer therapy activates the cGAS pathway in antigen-presenting cells such as dendritic cells, leading to the activation of antitumor immunity [17]. In addition, cGAS or STING agonists have been attracting attention because of their potential for enhancing antitumor immunity [15]. Taken together, these results suggest that the activation of the cGAS pathway in immune cells is valuable for cancer treatment, and that the cGAS pathway is a potential therapeutic target for the induction of antitumor effects in cancer therapy. However, the expression and function of cGAS in cancer cells are not fully understood. In particular, the role of cGAS in the response of cancer cells to treatment remains unclear.

In this study, we first investigated the expression of cGAS in solid tumors and its relationship with the prognosis of cancer patients using in silico analysis. We found that the expression of cGAS in head and neck squamous cell carcinoma (HNSCC) cells was high among solid tumor cell lines, and that HNSCC patients with high levels of cGAS in The Cancer Genome Atlas (TCGA) cohorts had a poor outcome. We analyzed the function and related mechanisms of cGAS in the response of HNSCC cells to radiation in vitro, because radiotherapy is one of the treatments most commonly used for HNSCC. cGAS knockdown increased the radiosensitivity of HNSCC cells, while the activation of cGAS increased the radioresistance of HNSCC cells. These results strongly suggest that the cGAS pathway regulates the radioresistance of HNSCC cells. We hope that this study will provide information helpful for improving the radiotherapy of HNSCC.

## 2. Materials and Methods

### 2.1. Bioinformatics and Data Analysis

Data about cGAS expression in solid tumor cell lines was downloaded from the Cancer Cell Line Encyclopedia (https://portals.broadinstitute.org/ccle, accessed on 12 September 2019) and reanalyzed. The levels of cGAS in tumors and adjacent normal tissues were compared using Cancer RNA-Seq Nexus (http://syslab4.nchu.edu.rw/, accessed on 12 September 2019). cBioPortal for Cancer Genomics (https://www.cbioportal.org/accessed on 12 September 2019) was used to analyze the association between the mRNA levels of cGAS and the survival time of patients with HNSCC, pancreatic cancer, and esophageal cancer in the TCGA cohort. The boundary between high and low expression levels was defined as the mean expression level plus one standard deviation.

### 2.2. Reagents

Annexin V Binding Buffer and FITC-linked annexin V (annexin V-FITC) was purchased from BioLegend (San Diego, CA, USA). Propidium iodide (PI) was purchased from Sigma-Aldrich (St. Louis, MO, USA). Anti-human phospho-IRF3 antibody (#37829), anti-human cGAS (#D1D3G) antibody (Biotinylated) (#66546), anti-human p21 antibody (#2947), β-actin antibody (#4967), Streptavidin-horseradish peroxidase (HRP) (#3999), anti-rabbit IgG HRP-linked antibody (#7074), and anti-mouse IgG HRP-linked antibody (#7076) were purchased from Cell Signaling Technology Japan, K.K. (Tokyo, Japan). ISD Control/LyoVec^TM^ and ISD/LyoVec^TM^ were purchased from InvivoGen (San Diego, CA, USA). Ambion Silencer^®^ Select pre-designed siRNA against the gene encoding cGAS (ID: s41748), the gene encoding CDKN1A (p21) (ID: s416), and Silencer^®^ Select Negative #1 Control siRNA (ID: 4390843) were purchased from Invitrogen (Thermo Fisher Scientific, Inc., Waltham, MA, USA).

### 2.3. Cell Culture and Treatment

Human HNSCC cell lines SAS and Ca9-22 were obtained from the RIKEN Bio-Resource Center (Tsukuba, Japan). Both cell lines were maintained in high glucose Dulbecco’s modified Eagle medium (DMEM; Wako Pure Chemical Industries, Ltd., Osaka, Japan) supplemented with 10% heat-inactivated fetal bovine serum (FBS, Sigma-Aldrich) and 1% penicillin/streptomycin (P/S, Wako Pure Chemical Industries). Cells were cultured at 37 °C under a humidified atmosphere of 5% CO_2_/95% air.

Cells were seeded at 1.0 × 10^5^ cells on 35 mm culture dishes (Sumitomo Bakelite Co., Ltd., Tokyo, Japan) or 2.0 × 10^5^ cells on 60 mm culture dishes (Sumitomo Bakelite Co., Ltd.), incubated for 6 h for adhesion, and then irradiated with X-rays. After four days of culture at 37 °C under a humidified atmosphere of 5% CO_2_/95% air, the cells were harvested using 0.25% trypsin-ethylenediaminetetraacetic acid (Wako Pure Chemical Industries Ltd.), and the number of viable cells was counted using trypan blue dye exclusion assays. These cells were then used for subsequent analyses.

### 2.4. siRNA Transfection

Cells were seeded in 24-well plates (4 × 10^4^ cells; BD Falcon; BD Biosciences, Franklin Lakes, NJ, USA) and transfected with siRNA using Lipofectamine^®^ RNAiMAX (Invitrogen; Thermo Fisher Scientific, Inc.), as previously reported [18]. The final concentration of each siRNA was 10 nM, except for 20 nM control siRNA for the simultaneous transfection of siRNAs targeting cGAS and p21. After transfection for 48 h, the cells were harvested and used for subsequent analyses.

### 2.5. In Vitro X-ray Irradiation

X-ray irradiation was performed using an X-ray generator (MBR-1520R-3; Hitachi Medical Corporation, Tokyo, Japan), as previously reported [19]. A dose rate of 0.99–1.03 Gy/min was monitored by placing a thimble ionization chamber next to the sample during irradiation.

### 2.6. Clonogenic Survival Assay

Cells seeded in 35 mm culture dishes were incubated for 6 h to allow adherence to the dishes, and were afterward irradiated with X-rays. In an experiment to test the effect of the cGAS agonist ISD/LyoVec^TM^ on the radiosensitivity of HNSCC cells, 500 ng/mL ISD Control/LyoVec^TM^ or ISD/LyoVec^TM^ was added to the culture medium 1 h before irradiation. After X-ray irradiation, the cells were cultured for about 24 h. After culturing, the cells were harvested, and appropriate cell numbers were seeded on 60 mm culture dishes for clonogenic survival assay. The cells were cultured for 8–12 days, fixed with methanol, and stained with Giemsa solution (Wako Pure Chemical Industries Ltd.). Experiments were performed in triplicate. Colonies containing more than 50 cells were counted. The surviving fraction was calculated using GraphPad Prism 9 (GraphPad Software, Inc., San Diego, CA, USA), as previously reported [20].

### 2.7. SDS-PAGE and Western Blotting

SDS-PAGE analysis and western blotting were performed as previously reported [21]. The following primary antibodies were used: Anti-cGAS antibody (1:3000), anti-p-IRF3 antibody (1:3000), anti-p21 antibody (1:3000), and anti-β-actin antibody (1:4000). The following secondary antibodies were used: Streptavidin-HRP (1:10,000), HRP-linked anti-rabbit IgG antibody (1:10,000), or HRP-linked anti-mouse IgG antibody (1:10,000). The antigens were visualized using the Clarity^TM^ Western ECL Substrate (Bio-Rad Laboratories Inc., Hercules, CA, USA). Stripping Solution (Wako Pure Chemical Industries, Ltd.) was used for blot stripping.

### 2.8. Mitotic Catastrophe

Cells (5 × 10^4^ cells) seeded on slide chamber (Iwaki, Tokyo, Japan) were incubated for 6 h, and then irradiated with X-rays. After four days of culture, the cells were washed with PBS(-) and fixed with 4% paraformaldehyde/PBS(-) for 5 min at room temperature. The cells were washed three times with PBS(-) and treated with 1% TritonX-100/PBS(-) for 20 min at room temperature. After three washes with PBS(-), the slides were sealed with Antifade Mounting Medium with DAPI (VECTASHIELD, LSBio). The slides were photographed using a fluorescence microscope (IX71, Olympus, Tokyo, Japan), and the nuclear morphology was evaluated. Cells with abnormal mitotic nuclear features such as micronuclei, multilobular nuclei, and fragmented nuclei were scored as cells in mitotic catastrophe [22]. More than 100 cells were counted for each condition.

### 2.9. Detection of Apoptosis

Apoptosis was analyzed using annexin V-FITC and PI staining, as previously reported [23]. The fluorescence intensity of annexin V-FITC and PI were analyzed using flow cytometry (Cytomics FC500 with CXP software ver.2; Beckman-Coulter, Inc., Brea, CA, USA). Annexin V^+^ cells (sum of annexin V^+^/PI^−^ and annexin V^+^/PI^+^ cells) were defined as apoptotic cells.

### 2.10. Analysis of SA-β-gal Activity

SA-β-gal activity was analyzed using the Cellular Senescence Detection Kit—SPiDER-βGal (DOJINDO, Kumamoto, Japan) according to the manufacturer’s instructions. In brief, after treatment, cells were washed with HBSS (Sigma-Aldrich) and incubated with HBSS containing 0.1% Bafilomycin A1 for 1 h at 37 °C under a humidified atmosphere of 5% CO_2_/95% air. After incubation, the cells were further incubated with HBSS containing 0.1% Bafilomycin A1 and 0.1% SPiDER-βGal for 30 min at 37 °C under a humidified atmosphere of 5% CO_2_/95% air. After washing twice with HBSS, the cells were harvested, washed with HBSS, and suspended in HBSS. The fluorescence intensity of the SPiDER-βGal was analyzed using a flow cytometer (Cytomics FC500 with CXP software ver.2).

### 2.11. Statistical Analysis

Data are presented as the mean ± SD of at least three independent experiments. Statistical analyses were performed using Excel 2016 software (Microsoft Corporation, Redmond, WA, USA) along with the add-in software Statcel 4 (OMS publishing Inc., Tokyo, Japan) or GraphPad Prism 9. A *p* value of <0.05 was considered to be statistically significant.

## 3. Results

### 3.1. Association between cGAS and Malignancies of HNSCC

**Figure 1:**
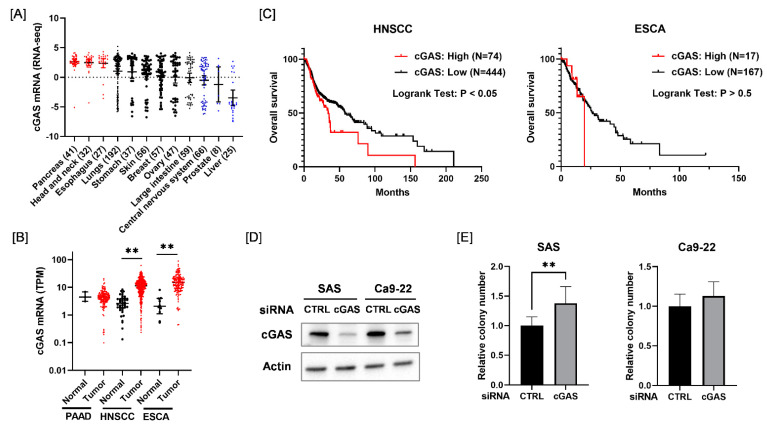
Relationship between the expression of cGAS mRNA and survival in each disease. (**A**) Expression levels of cGAS mRNA in each solid tumor cell line. (**B**) Black and red symbols indicate cGAS expression levels in adjacent normal tissues and tumor tissues, respectively. PAAD, pancreatic adenocarcinoma; HNSCC, head and neck squamous cell carcinoma; ESCA, esophageal carcinoma. (**C**) Relationship between the expression level of cGAS and patients’ overall survival. High cGAS expression was associated with poor overall survival of HNSCC patients in the TCGA cohorts. (**D**) HNSCC cells (SAS and Ca9-22) transfected with the control or cGAS siRNA were harvested, and cGAS protein expression was analyzed using western blotting. A representative image of an immunoblot is shown. Actin was used as the loading control. (**E**) The proliferative ability of cGAS knockdown cells was assessed by colony formation assay. ** indicates *p* < 0.01.

We first analyzed the expression of cGAS mRNA across different types of cancer cells from the Cancer Cell Line Encyclopedia. As shown in Figure 1A, cGAS mRNA levels were higher in pancreatic cancer, HNSCC, and esophageal cancer cell lines compared with other solid tumor cell lines. Therefore, we next analyzed the expression of cGAS mRNA in the tumor tissues of pancreatic cancer, HNSCC, and esophageal cancer and adjacent normal tissues using Cancer RNA-Seq Nexus [24]. The expression of cGAS mRNA in HNSCC and esophageal cancer in tumor tissues was significantly higher than that in normal tissues (Figure 1B). We also examined the association between the expression of cGAS mRNA and the overall survival of HNSCC and esophageal cancer patients using the cBioPortal for Cancer Genomics [25]. Kaplan–Meier analysis of the TCGA cohorts revealed that a high expression of cGAS was significantly correlated with a poor outcome for HNSCC patients, but not for esophageal cancer patients (Figure 1C). Since these results suggest that cGAS is associated with HNSCC among the solid tumors, we hereafter investigated the role of cGAS in HNSCC cells.

Considering the results of the in silico analysis, we expected that cGAS may promote the proliferation of HNSCC cells. However, cGAS knockdown HNSCC cells (SAS and Ca9-22) showed similar or greater proliferative ability compared with the control cells (Figure 1D,E). This result suggests that the cGAS does not promote the proliferation of SAS and Ca9-22.

### 3.2. Involvement of the cGAS Pathway in the Radiosensitivity of HNSCC Cells

**Figure 2:**
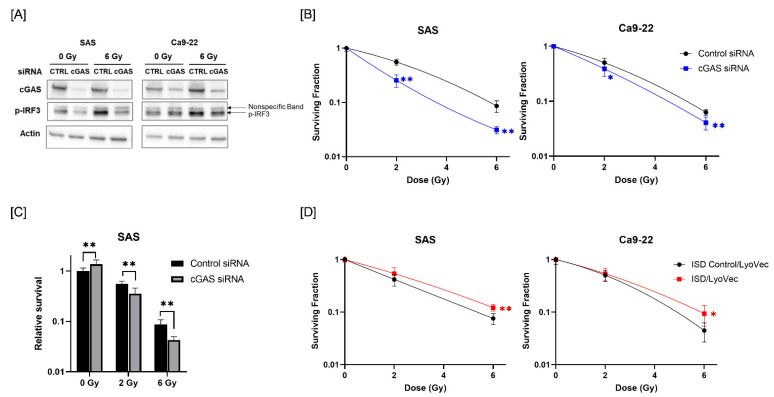
Effect of cGAS knockdown and agonist on the radiosensitivity of HNSCC cells. (**A**) HNSCC cells (SAS and Ca9-22) transfected with the control or cGAS siRNA were harvested and irradiated with 6 Gy X-rays. After 24 h (SAS) or 48 h (Ca9-22) of culture, the cells were harvested for western blot analysis. A representative image of an immunoblot is shown. Actin was used as the loading control. p-IRF3 indicates phosphorylated-IRF3. (**B**) cGAS knockdown HNSCC cells were irradiated with X-rays. After about 24 h of culture, the cells were harvested and seeded for colony formation assays. The results are shown as the surviving fraction of HNSCC cells. (**C**) Relative survival of SAS cells compared with non-irradiated cells transfected with control siRNA. (**D**) HNSCC cells treated with ISD Control/LyoVec^TM^ or ISD/LyoVec^TM^ for 24 h were irradiated with X-rays, and further cultured for 24 h. After culturing, the cells were harvested and seeded for colony formation assays. Results are shown as the surviving fraction of irradiated cells. * and ** indicate *p* < 0.05 and *p* < 0.01, respectively.

Since radiotherapy is one of the most commonly used treatments for HNSCC [26], we next analyzed the role of cGAS in the response of HNSCC cells to radiation. We first analyzed whether radiation activates the cGAS pathway in HNSCC cells. We focused on the phosphorylation of IRF3, which is a downstream factor of the cGAS pathway [27]. As shown in Figure 2A, the expression of phosphorylated-IRF3 (p-IRF3) was increased in HNSCC cells after X-ray irradiation, and cGAS knockdown suppressed the radiation-increased p-IRF3. These results suggest that ionizing radiation activates IRF3 through cGAS in HNSCC cells.

Next, we examined the involvement of cGAS in the radiosensitivity of HNSCC cells. As shown in Figure 2B, cGAS knockdown decreased the surviving fraction of irradiated HNSCC cells compared with a control siRNA group, thus indicating that there is a radiosensitizing effect of cGAS knockdown in HNSCC cells. Since cGAS knockdown of SAS cells showed high proliferative ability (Figure 1E), we analyzed the eventual antitumor effect of cGAS knockdown on irradiated SAS cells by calculating the survival rate against that of non-irradiated SAS cells transfected with control siRNA. The relative survival of X-irradiated SAS cells was significantly lower in the cGAS knockdown group than in the control group (Figure 2C).

We further examined the effect of the cGAS agonist ISD/LyoVec^TM^ on the radiosensitivity of HNSCC cells. As shown in Figure 2D, the surviving fraction following 6 Gy-irradiation was significantly higher in the ISD/LyoVec^TM^-treated group than in the ISD Control/LyoVec^TM^ group (Figure 2D). Collectively, these results indicate that cGAS is involved in the regulation of radiosensitivity in HNSCC cells.

### 3.3. Effect of cGAS Knockdown on Radiation-Induced Mitotic Catastrophe in SAS Cells

**Figure 3:**
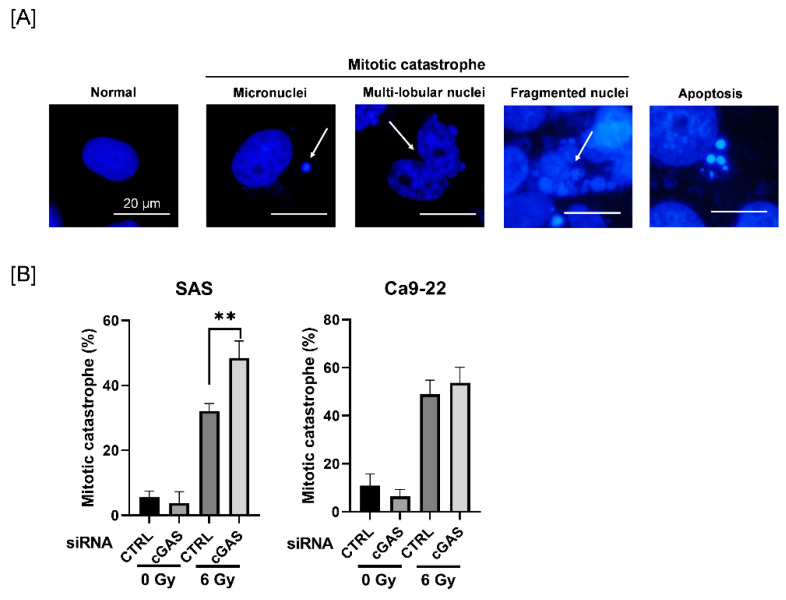
Effect of cGAS knockdown on radiation-induced mitotic catastrophe in HNSCC cells. (**A**,**B**) HNSCC cells transfected with the control or cGAS siRNA were harvested and treated with 6 Gy. After four days of culture, the cells were harvested for the analysis of mitotic catastrophe. (**A**) Representative pictures of mitotic catastrophe are shown. Micronuclei, multilobed nuclei, and fragmented nuclei were counted as indicating mitotic catastrophe. The arrows indicate the cells undergoing mitotic catastrophe. (**B**) The percentages of mitotic catastrophe cells are shown. ** indicate *p* < 0.01, respectively.

Since mitotic catastrophe, one of the forms of radiation-induced cell death, is primarily related to reproductive cell death caused by ionizing radiation [28], we next investigated the effect of cGAS knockdown on radiation-induced mitotic catastrophe in HNSCC cells. To identify mitotic catastrophe, the cells were analyzed for the presence of micronuclei, multilobular nuclei, and fragmented nuclei, not apoptosis (Figure 3A). As shown in Figure 3B, the percentage of mitotic catastrophe in irradiated SAS cells was significantly higher in the cGAS knockdown group than in the control group, whereas there was no significant difference between the control and the cGAS knockdown groups in Ca9-22 cells. These results suggest that cGAS prevents radiation-induced mitotic catastrophe, thus leading to radioresistance in SAS cells.

### 3.4. Effect of cGAS Knockdown on Radiation-Induced Apoptosis and Cellular Senescence in Ca9-22 Cells

Since cGAS knockdown produced no changes in radiation-induced mitotic catastrophe in Ca9-22 cells, we next investigated the effects of cGAS knockdown on radiation-induced apoptosis and cellular senescence in Ca9-22 cells, because apoptosis and cellular senescence are also known to contribute to reproductive cell death by radiation [29,30].

**Figure 4:**
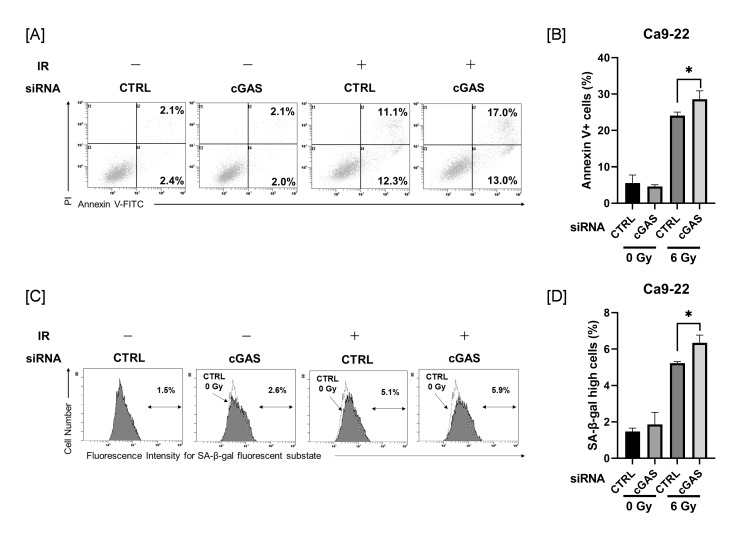
Effect of cGAS knockdown on radiation-induced apoptosis and high SA-β-gal activity in Ca9-22 cells. (**A**,**B**) cGAS knockdown Ca9-22 cells were irradiated with X-ray. After four days of culture, the analysis of apoptosis was performed. (**A**) Representative cytograms are shown. The inset numbers indicate the percentage of annexin V^+^/PI^−^ cells or annexin V^+^/PI^+^ cells. (**B**) The annexin V^+^ apoptotic cells induced by 6 Gy is shown. (**C**,**D**) X-irradiated Ca9-22 cells were cultured for four days and SA-β-gal activity was analyzed using the senescence β-Galactosidase Activity Assay Kit. Representative histograms of SA-β-gal activity in Ca9-22 cells are shown. The dotted line shows the results of non-irradiated cells, and the inset numbers show the percentage of cells with high SA-β-gal activity. (**D**) The percentage of SA-β-gal high-activity cells is shown. * indicates *p* < 0.05.

As shown in Figure 4A, analysis of apoptosis using annexin V staining revealed that irradiation with 6 Gy increased the proportion of annexin V^+^ apoptotic HNSCC cells. The proportion of annexin V^+^ in 6 Gy-irradiated Ca9-22 cells was significantly higher in the cGAS knockdown group than in the control group (Figure 4B). The proportion of Ca9-22 cells with high SA-β-gal activity, one of the hallmarks of cellular senescence, was increased by 6 Gy-irradiation, and the proportion in the cGAS knockdown group was significantly higher than that in the control group (Figure 4C,D). These effects induced by cGAS knockdown were not observed in SAS cells (Figure S1). These results suggest that cGAS controls the radioresistance of Ca9-22 cells through the regulation of radiation-induced apoptosis and/or cellular senescence.

### 3.5. Effect of Double Knockdown of cGAS and p21 on Radiation-Induced Cellular Senescence, Apoptosis, and Mitotic Catastrophe in Ca9-22 Cells

**Figure 5:**
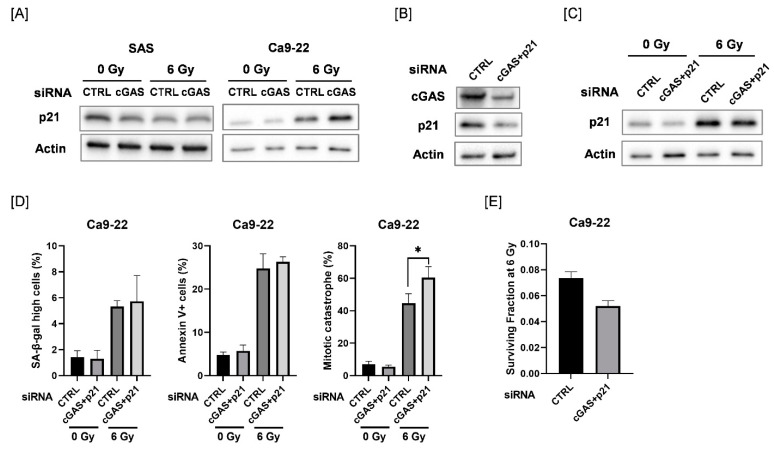
Involvement of p21 in the cGAS-mediated radiation response of Ca9-22 cells. (**A**) HNSCC cells (SAS and Ca9-22) transfected with the control or cGAS siRNA were harvested and irradiated with 6 Gy X-rays. After four days of culture, the cells were harvested for western blot analysis. A representative immunoblot image is shown. Actin was used as the loading control. (**B**) Ca9-22 cells transfected with control or cGAS and p21 siRNA were harvested. After 24 h of culture, the cells were harvested for western blot analysis. A representative immunoblot image is shown. Actin was used as the loading control. (**C**,**D**) Ca9-22 cells transfected with the control or cGAS and p21 siRNA were irradiated with 6 Gy X-rays and cultured for four days. The cells were harvested for western blotting (**C**) and analysis for SA-β-gal activity, apoptosis, and mitotic catastrophe (**D**). (**C**) A representative immunoblot image is shown. Actin was used as the loading control. (**D**) The percentages of SA-β-gal high-activity, annexin V^+^ apoptotic cells, and mitotic catastrophe are shown. * indicates *p* < 0.05. (**E**) Ca9-22 cells transfected with control or cGAS and p21 siRNA were irradiated with 6 Gy X-rays. After about 24 h of culture, the cells were harvested and seeded for colony formation assays. The results are shown as the surviving fraction at 6 Gy. The representative result is shown. Similar results were obtained in two different experiments. The data are mean ± SD of triplicate.

We explored the mechanisms causing the difference in cGAS-mediated cellular responses between SAS and Ca9-22 cells. We focused on p21, because p21 is involved in the regulation of mitotic catastrophe as well as in cellular senescence [31,32]. As shown in Figure 5A, the protein expression of p21 was increased after 6 Gy-irradiation in Ca9-22 cells, and radiation-increased p21 expression was further increased by cGAS knockdown. The upregulation of p21 after irradiation was not observed in SAS cells. We further investigated the effect of simultaneous knockdown of cGAS and p21 in Ca9-22 cells on cellular senescence, apoptosis, and mitotic catastrophe after irradiation (Figure 5B–D and Figure S2). Although the simultaneous transfection of cGAS and p21 siRNA did not completely inhibit the radiation-increased p21 expression, additional knockdown of p21 could diminish the upregulation of p21 by cGAS knockdown in irradiated Ca9-22 cells (Figure 5C). When both cGAS and p21 siRNA were simultaneously transfected, the significant increase in the proportion of irradiated cGAS knockdown cells with high SA-β-gal activity and apoptosis disappeared while mitotic catastrophe in the irradiated Ca9-22 cells was significantly increased (Figure 5D). These results suggest that p21 is involved in the difference in cGAS-mediated cellular responses between SAS and Ca9-22 cells. In addition, the simultaneous transfection of cGAS and p21 siRNA also increased the sensitivity to 6 Gy irradiation in Ca9-22 cells (Figure 5E), thus implying that the mitotic catastrophe is involved in the enhancement of radiosensitivity observed in Ca9-22 cells transfected with cGAS and p21 siRNA.

## 4. Discussion

In addition to the originally identified role of cGAS as a response to pathogens, recent studies have shown the importance of the cGAS pathway in cancer therapy, via the induction of antitumor immunity [17,33], and the relationship between cGAS levels and the prognosis of cancer patients [16]. However, the role of cGAS in cancer cells remains to be elucidated. In this study, in silico analysis indicated an association of cGAS with HNSCC, and in vitro analysis revealed the involvement of cGAS in the radioresistance of HNSCC cells.

The inhibition of the growth of cancer cells is an important factor in cancer therapy. Unfortunately, cGAS knockdown increased the proliferation of non-irradiated SAS cells, although it effectively suppressed the proliferation of irradiated SAS cells. It has been reported that the loss of the cGAS gene promotes the proliferation of colon cancer cells in vivo [34]. Chen et al. showed that cGAS-deficient human fibroblast BJ cells and osteosarcoma U2OS cells showed accelerated replication fork progression, leading to hyperproliferation [35]. Therefore, there is a possibility that cGAS-mediated regulation of replication fork progression may regulate the proliferation of non-irradiated SAS cells.

DNA damaging agents such as doxorubicin are known to induce the phosphorylation of IRF3, one of the downstream molecules of cGAS [27]. It has also been reported that radiation induces IFN-beta in several types of cancer cells such as breast cancer cells, and the knockdown of cGAS or STING suppressed this effect [33]. In keeping with these reports, the result of the present study showed that ionizing radiation induced the phosphorylation of IRF3 in HNSCC cells, and that this phosphorylation was partly suppressed by cGAS knockdown, thus suggesting that ionizing radiation activates the cGAS pathway in HNSCC cells. It has been reported that exosomes containing tumor-derived DNA from irradiated breast cancer cells activate the cGAS-STING pathway in dendritic cells [36]. Therefore, it is possible that exosomes containing tumor-derived DNA can induce the activation of the cGAS-STING pathway in irradiated HNSCC cells. Of course, there is a possibility that cGAS detects the self-DNA of irradiated cells, because a recent report demonstrated that cGAS is translocated to chromosomes in mitosis [37].

Chen et al. recently reported that cGAS-deficient BJ and U2OS cells have increased sensitivity to DNA damaging agents including ionizing radiation [35]. In agreement with their report, in our study, cGAS knockdown HNSCC cells showed increased radiosensitivity. Together, these results suggest that cGAS protects not only normal cells, but also cancer cells from ionizing radiation, and that cGAS may be a potential target to enhance the radiosensitivity of cancer cells in radiation therapy.

We showed that cGAS knockdown increased radiation-induced mitotic catastrophe in SAS cells. Since the induction of mitotic catastrophe is closely associated with high radiosensitivity [22,38], it is likely that the increase in mitotic catastrophe in irradiated SAS cells is one of the mechanisms by which cGAS knockdown induced the radiosensitizing effect observed in SAS cells. Mitotic catastrophe occurs due to various factors such as premature or inappropriate entry of cells into mitosis, destabilization of the replication fork, and defects in cell cycle checkpoints [39,40]. Therefore, to clarify the mechanisms of the cGAS-mediated regulation of mitotic catastrophe, the effects of cGAS knockdown on factors related to mitotic catastrophe should be analyzed in a future study.

cGAS knockdown increased the radiosensitivity of Ca9-22 cells without enhancing radiation-induced mitotic catastrophe, thus suggesting that the mechanisms of the radiosensitizing effects of cGAS knockdown in HNSCC cells depend on the cell line. We found that cGAS knockdown increased the number of apoptotic and senescent cells (i.e., high SA-β-gal activity cells) following irradiation of Ca9-22 cells. Since both mitotic catastrophe and apoptosis and cellular senescence are related to cellular radiosensitivity [41], the enhancement of apoptosis and senescence by cGAS knockdown may contribute to the radiosensitizing effect of cGAS knockdown in Ca9-22 cells. In addition to mitotic catastrophe, p21 is involved in cGAS-mediated apoptosis and cellular senescence in irradiated Ca9-22 cells. Since p21 is a well-known factor inducing cellular senescence [42], it is not surprising that p21 knockdown attenuated the proportion of cGAS knockdown-increased senescent cells in irradiated Ca9-22 cells. However, although p21 is generally considered to produce anti-apoptotic effects [43], the results of this study suggested a pro-apoptotic effect of p21, because p21 knockdown attenuated the cGAS knockdown-increased apoptosis in irradiated Ca9-22 cells. Some previous reports have shown a pro-apoptotic function of p21. For example, Ghanem et al. reported that p21 is pro-apoptotic in granulopoiesis [44]. It has been reported that overexpression of p21 enhances cisplatin-induced apoptosis in human ovarian carcinoma cells [45].

**Figure 6:**
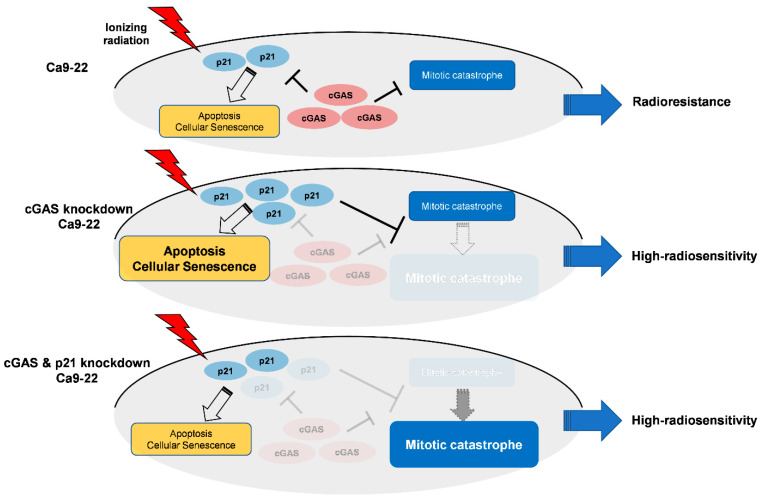
Hypothetical model of the roles of cGAS and p21 in irradiated Ca9-22 cells.

Intriguingly, double knockdown of cGAS and p21 diminished cGAS knockdown-facilitated apoptosis and senescence as well as p21 expression in irradiated Ca9-22 cells (Figure 5C,D), while it enhanced mitotic catastrophe (Figure 5D), thus suggesting the key roles of p21 to determine the cell death mode in cGAS-mediated radiation responses in Ca9-22. Figure 6 shows a hypothetical model of the roles of cGAS and p21 in irradiated Ca9-22 cells. In cGAS knockdown cells, radiation-induced p21 further increases. The upregulated p21 can promote apoptosis and cellular senescence in irradiated cells, while it prevents an increase in mitotic catastrophe. The role of p21 to suppress radiation-induced mitotic catastrophe is consistent with the report by Bunz et al. [46]. In cGAS and p21 knockdown cells, cGAS knockdown-facilitated p21 expression in irradiated cells is diminished. As a result, in double knockdown cells, cGAS knockdown-facilitated apoptosis and senescence are diminished, while an increase in mitotic catastrophe by cGAS knockdown phenotypically appears because a suppression of mitotic catastrophe through cGAS knockdown-facilitated p21 disappears. It would be interesting to analyze the mechanisms underlying the different responses of p21 in SAS and Ca9-22 cells after irradiation in a future study.

Yang et al. showed that the loss of the cGAS gene inhibited radiation-induced senescence in mouse embryonic fibroblasts cells, mouse melanoma B16F10, and BJ cells [16]. In contrast, no significant suppression of radiation-induced senescent cells was observed in cGAS knockdown Ca9-22 cells in this study. Although the reason for the discrepancy between their and our report remains unclear, one explanation may be differences in the type of cell lines used in the studies. For example, the tumor suppressor gene TP53 is involved in the induction of cellular senescence [47]. All cell lines used in Yang’s report had wild type TP53, whereas TP53 was mutated in both the SAS and Ca9-22 cell lines (refer to the review report of [18]).

Akin to cGAS, retinoic acid inducible gene-I-like receptors (RLR) are pattern recognition receptors, and can induce type I IFN [48]. Our previous report showed that the RLR agonist exerts radiosensitizing effects against human lung cancer cells [19]. We originally speculated that the cGAS agonist would also have a radiosensitizing effect, because type I IFN is known to show a radiosensitizing effect against various tumor cells including HNSCC cells [49]. However, contrary to our speculation, the cGAS agonist induced radioresistance in HNSCC cells. This result suggests the possibility that the cGAS agonist induces radioresistance in HNSCC cells in a type I IFN-independent manner. Since type I IFN is known to exert antitumor effects [50], further study clarifying the mechanisms of cGAS agonist induced radioresistance will lead to more effective radiation therapy for HNSCC cells.

## 5. Conclusions

Here, we demonstrated that cGAS is highly expressed in HNSCC cells and related to poor prognosis for HNSCC patients. We also showed that the cGAS pathway regulates the radioresistance of HNSCC cells. However, the molecular mechanisms of cGAS-mediated radioresistance remain unknown. Further studies on the clarification of molecular mechanisms and in vivo experiments are needed to determine the eventual role of cGAS in HNSCC cells.

## Data Availability

The data presented in this study are available in the article.

## Supplementary Materials

The following supporting information can be downloaded at: https://www.mdpi.com/article/10.3390/cells11091434/s1, Figure S1: Effect of cGAS knockdown on radiation-induced apoptosis and high SA-β-gal activity in SAS cells. Figure S2: Effect of double knockdown of cGAS and p21 on radiation-induced high SA-β-gal activity in Ca9-22 cells.
