# Supplementary material for: cGAS Regulates the Radioresistance of Human Head and Neck Squamous Cell Carcinoma Cells
Source: Cells. 2022 Apr 23;11(9):1434. doi: 10.3390/cells11091434 (PMC9101626; doi:10.3390/cells11091434)
Supplement: Supplementary file 1 [file cells-11-01434-s001.zip › cells-1659702-supplementary.pdf]

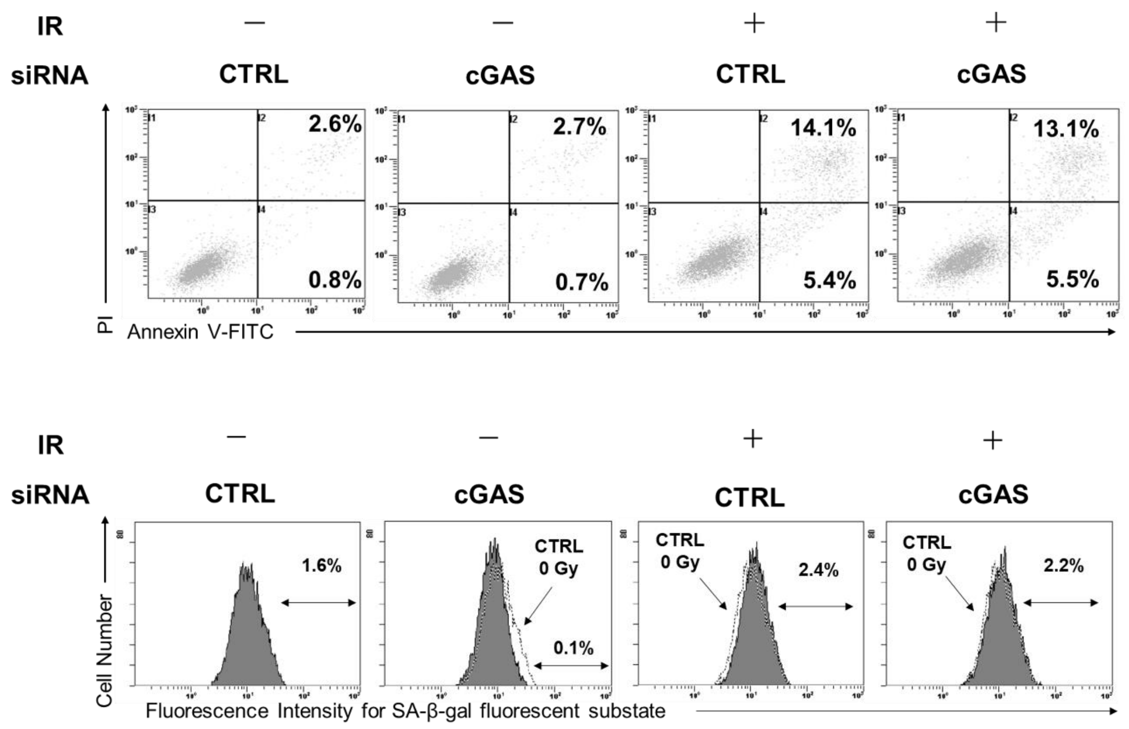

Figure S1. Effect of cGAS knockdown on radiation-induced apoptosis and high SA-β-gal activity in SAS cells. [A] cGAS knockdown SAS cells were irradiated with X-ray. After four days of culture, the cells were harvested for the analysis of apoptosis. [A] Representative cytograms are shown. The inset numbers indicate the percentage of annexin V^+/PI^- cells or annexin V^+/PI^+ cells. [B] X-irradiated SAS cells were cultured for four days and SA-β-gal activity was analyzed using senescence β-Galactosidase Activity Assay Kit. Representative histograms of SA-β-gal activity in SAS cells are shown. The dotted line shows the results of non-irradiated cells, and the inset numbers show the percentage of cells with high SA-β-gal activity.

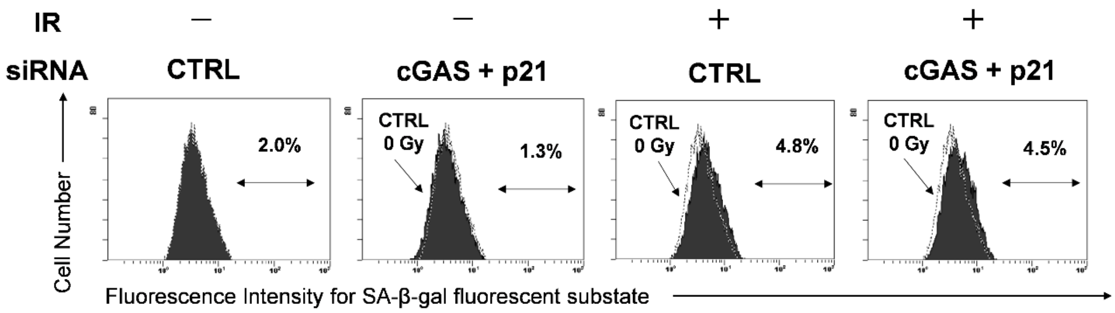

Figure S2. Effect of double knockdown of cGAS and p21 on radiation-induced high SA-β-gal activity in Ca9-22 cells. Ca9-22 cells transfected with control or cGAS and p21 siRNA were irradiated with 6 Gy X-rays and cultured for four days. The cells were harvested for analysis for SA-β-gal activity. Representative histograms of SA-β-gal activity in Ca9-22 cells are shown. The dotted line shows the results of non-irradiated cells, and the inset numbers show the percentage of cells with high SA-β-gal activity.
